# Monitoring Heart Disease and Diabetes with Mobile Internet Communications

**DOI:** 10.1155/2012/195970

**Published:** 2012-11-18

**Authors:** David Mulvaney, Bryan Woodward, Sekharjit Datta, Paul Harvey, Anoop Vyas, Bhaskar Thakker, Omar Farooq, Robert Istepanian

**Affiliations:** ^1^School of Electronic, Electrical and Systems Engineering, Loughborough University, Leicestershire LE11 3TU, UK; ^2^Instrument Design Development Centre, The Indian Institute of Technology Delhi, Hauz Khas, New Delhi 110016, India; ^3^Department of Electronics Engineering, Aligarh Muslim University, Aligarh 202002, India; ^4^Faculty of Science, Engineering and Computing, Kingston University, London KT1 2EE, UK

## Abstract

A telemedicine system is described for monitoring vital signs and general health indicators of patients with cardiac and diabetic conditions. Telemetry from wireless sensors and readings from other instruments are combined into a comprehensive set of measured patient parameters. Using a combination of mobile device applications and web browser, the data can be stored, accessed, and displayed using mobile internet communications to the central server. As an extra layer of security in the data transmission, information embedded in the data is used in its verification. The paper highlights features that could be enhanced from previous systems by using alternative components or methods.

## 1. Introduction

Telemedicine is literally medicine from a distance and is synonymous with a telecommunications network for the transmission of medical information. The premise is that patients can be monitored away from a hospital or health centre, thereby accessing medical care that would otherwise be unavailable, given the time and cost of travelling. From a clinical standpoint remote monitoring allows patients to record their readings in a more relaxed home environment, rather than undergo the possible stress of travelling for a personal consultation. This is now a mature technology and the literal requirement for large distances has now diversified to include groups within local networks such as a hospital ward, a home network surrounding the patient and using mobile devices specifically. Diversified branches of telemedicine are generally referred to as e-health (electronic health), u-health (ubiquitous health), and m-health (mobile health) systems, amongst others.

The fundamental parts of the telemedicine system are the measuring devices (e.g., instruments or sensors), a device or subcomponent (e.g., mobile phone, computer) to format the readings for a communications link, a clinic server to which the data is transmitted, a database for storing the data, and a display of the data obtained from the server. A typical system uses a dedicated program on a computer device (e.g., laptop, iPad) to obtain the patient's readings and a web browser for other users to access previous readings. The many types of conditions monitored include heart monitoring using an electrocardiograph (ECG) [1], type 1 and type 2 diabetes [2, 3], psoriasis [4], shoulder surgery [5], and chronic obstructive pulmonary disease (COPD) [6]. 

Since this technology has been an active research area for at least twenty years, and many systems have been implemented for research and commercial use, our UK-India Education and Research Initiative (UKIERI) project [7] has a remit of implementing a telemedicine system specifically for cardiac and diabetic monitoring using mobile telecommunications networks in India. The components of the system are now in place and technical trials are underway with clinical trials to follow. 

By exploiting previous work, we identified and addressed some areas of a typical system that would require enhancement to provide the level of monitoring envisaged. In particular these were

- to develop a network of wireless sensor nodes worn by the patient to record typical vital signs and to be compatible with our current research of obtaining diagnostic information from the analysis of a patient's wrist pulse. To transmit between nodes, a low-power, high data-rate protocol has been used as an alternative to ZigBee and Bluetooth;
- to identify specific measurements to be recorded by the system to provide indicators for trend analysis and possible early warning of complications. Besides vital signs from the sensors, readings representing a patient's well-being from other instruments are combined into a comprehensive set of measured parameters;
- to allow greater use of web browsers, and in particular standard mobile device web browsers, without the need to install a specific device application. Using newer browser features, the system's web pages can use a browser in a similar way to previous systems' downloaded applications;
- to enhance the security of transmitted patient data by embedding identification information into the contents of the downloaded file, thus verifying that it has not been tampered with.

The rest of this paper is structured as follows: Section 2 details the system implemented and in particular how it addressed the points above; Section 3 features the results of technical trials; Section 4 is a discussion on the findings and the scope for future work.

## 2. System Architecture

**Figure 1:**
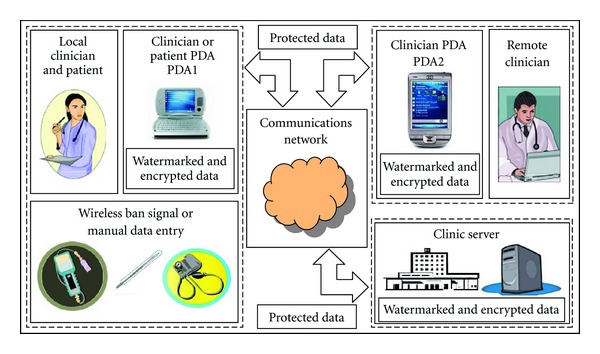
Schematic of the telemedicine system architecture.

The system architecture is shown in Figure 1. The patient end comprises wireless medical sensor nodes, a mobile device (PDA), and other medical instruments without communication capability. An internet link over Wi-Fi or 3 G connects the PDA to the server and database. Other users can then access the data on the web browser of a PC or mobile device.

### 2.1. System Overview

The sensor nodes are worn by the patient as a body area network (BAN) and are arranged as a master and multiple slaves. The slave nodes communicate with the master node via wireless transceivers. The master node is a similar device but also has a Bluetooth transceiver to link to the PDA. Since Bluetooth is ubiquitous to such mobile devices, this part of the telemetry link is predetermined. The link between the wireless nodes has more options, ranging from Bluetooth or ZigBee to proprietary protocols. Our nodes use a proprietary Nordic 2.4 GHz transceiver used for low-power, long battery life applications such as PC components and medical sensors. This allows one master node to communicate with a star network of up to six slave nodes. Although such measurements as temperature, respiration, and ECG lend themselves to be acquired electronically with no invasive procedure (although it could be argued that certain sites used for body temperature measurement are invasive to some extent), the measurement of blood sugar is still largely performed using blood samples directly. Although some of such devices have wireless capabilities, most do not, so it is useful to include wireless-free instruments in the BAN to allow the patient to read and record values directly. We used a wireless-free LifeScan OneTouch Ultra 2 glucose meter in the evaluation. Hence our system is designed for acquiring readings from both wireless and standard instruments.

The patient PDA has Bluetooth for the node communication and also 3G/EDGE/GPRS mobile phone and Wi-Fi capabilities for internet access. Its Windows Mobile operating system has similar capabilities to a Java-based system on other comparable devices. The PDA application is used to acquire, store, upload, download, and display medical readings. Using Bluetooth, it can configure and receive data from the master node to be stored locally to the PDA for uploading later. For standard instruments, displayed values are entered into the application's spreadsheet forms, which are designed for the purpose of monitoring specific parameters associated with cardiac and diabetic conditions. Some colour coding of spreadsheet values allows a quick visual highlight of problem areas. The mobile web browser on the PDA cannot control the Bluetooth link, but the user can still acquire, upload, download, and display medical readings using the system's web pages. The data acquisition uses the same spreadsheet interface as the application, allowing readings from the standard instruments to be entered. 

The server and database platforms used to store the patient measurements are Apache, PHP, and MySQL. The server and database are situated in England and can readily be accessed by the project partners in India. To increase the scope of participants to view the data, any clinician (such as a specialist located in a different country) or other user (perhaps wishing to view their condition periodically) should be able to access the data without having to download a specific software application. By taking into account the lesser capabilities of a mobile device web browser compared to a PC browser, our system allows a browser on a PC or mobile to be used to search and view patient data as text or images with a similar capability to the PDA application. 

### 2.2. Wireless Sensor Nodes

The prototype wireless slave and master nodes share a common design with power supply, signal acquisition, microcontroller, and one or two wireless transceivers, respectively.

#### 2.2.1. Slave Nodes

These are worn by the patient and connected to the measurement transducer with a wired link. To contribute to a prolonged node battery life, the node's microcontroller unit (MCU) is an MSP430F2618E mixed signal device from Texas Instruments [8]. This is a 16 MHz 16-bit RISC ultra-low-power MCU including 12-bit ADC, four universal serial communication interfaces (USCI), and direct memory access (DMA) controller. Three slave nodes are used to measure ECG, temperature, and respiration respectively. A fourth node, to acquire the signals for wrist pulse analysis of a patient, is currently being researched and uses noninvasive pressure sensors to measure the pressure over the radial artery of both wrists. Wrist pulse analysis is used in the Indian Ayurveda system for health state diagnosis [9]. The transducer signal is taken through an analogue signal conditioning stage, then to the ADC of the MCU. The MCU's DMA facility allows the fast transfer of data between memory addresses and this is used in the digital filtering stage. The ADC output is filtered with the digital filter and sent to the Nordic transceiver by a serial peripheral interface (SPI) on a USCI port. The Nordic transceiver sends the data wirelessly to the master node.

#### 2.2.2. Master Node

This is also worn by the patient and has the same design as the slaves but also has a Bluetooth module to communicate with the patient PDA. The Nordic transceiver is placed in receive mode and listens for incoming data from each slave node polled in turn by the master. The data are read from the transceiver by the MCU on a USCI port and sent to the Bluetooth module on a second USCI port. The Bluetooth module is a KC Wirefree KC-21 device [10] supporting the Bluetooth serial port profile (SPP) and driven by AT commands from the MCU [11]. Once the master node is Bluetooth-paired with the patient PDA, the PDA initiates a connection by opening its virtual serial port to the node and communications can begin.

#### 2.2.3. Node Nordic Transceiver

The main consideration in selecting the Nordic transceivers is the node's size and battery life. The wireless transceivers on the patient nodes use the international ISM 2.4 GHz band, which is a common option for BANs. It also allows the use of a small antenna and in turn a small node for placing around the body. The problem with a wearable node is RF losses between nodes due to absorption through the body [15, 16]. To mitigate these losses, one option is for the nodes to increase their output power at the risk of reduced battery life and increased localised radiation absorption. Another option is to use the nodes' medium access control (MAC) protocol so that the transceiver is only powered when necessary. The latter option has generated research into the design of the node network topology and also the MAC protocols. Examples for sending node data directly to a master node (single hop) include [17], and nodes sending on data from other nodes (multihop) [18]. Such strategies are designed to allow the nodes to be on reduced power for most of the time to conserve battery life, but still use low power transmissions when required.

The options for 2.4 GHz transceiver devices with suitable MACs include Bluetooth low energy technology [19], ZigBee [20], ShockBurst [21], and ANT [22]. Classic Bluetooth has also been used in BANs [23], although it is not targeted at small battery cell applications. Low energy Bluetooth devices were not available for this project, so some comparisons between the others are shown in Table 1.

The nodes use the nRF24L01 ShockBurst transceivers from Nordic Semiconductor [13]. These have a higher bit rate than ZigBee (up to 2 Mbps rather than up to 250 kbps for ZigBee), the MAC protocol is simple to implement, and six nodes are adequate for the network, so multihop is not required. The ShockBurst network topology consists of a 6-point star with the master node at the centre of the slave nodes. The master node communicates through “data pipes,” which are assigned unique addresses. By setting a slave node to the same address as a data pipe, the master can communicate with each node. To help mitigate data collisions between similar nodes nearby, each node in a particular network is configured to use the same frequency channel from the 83 available between 2.4–2.4835 GHz. 

The MAC protocol also has an automatic data packet assembly and autorepeat function for lost packets. Each packet comprises 1 byte of preamble, 3 to 5 bytes holding the pipe address, 9 control bits including a 2-bit packet number, up to 32 bytes of payload and 2 bytes of CRC covering the address, control, and payload. After a slave node transmits a packet, it listens for an acknowledgement from the master node. If the acknowledgement is not received, the packet is retransmitted. The 2-bit packet number is incremented for each new packet so the master node can detect repeated packets. The transceiver transmits new data as soon as its controlling MCU instructs it to; the delivery of the data is then left up to the transceiver, which can report back any problems via registers. As soon as the master node receives a packet with a valid address, it checks the CRC, transmits an acknowledgment to the same address, and stores the payload in a register for its controlling MCU to read. In case of repeated packets, the 2-bit packet count will be unchanged so the payload is not updated. By delegating the packet delivery to the transceiver's protocol it frees up the node MCU for the signal acquisition task.

#### 2.2.4. Node Bluetooth Transceiver

The Bluetooth link between the master node and the patient PDA uses a data exchange protocol using the SPP of the KC-21 module, which is a wire-replacement profile for delivering bytes. In “command mode,” the KC-21 can be configured with AT commands to set up the serial link with settings, including baud rate and parity. In “bypass mode” any data received by the module is sent over the SPP acting as a virtual cable to the PDA. Separate protocols for the node network configuration and for receiving measurement data are used. The network is configured on the PDA application by selecting which slave nodes to receive data from and their respective sample rates. For receiving the data, the protocol has less overhead than the configuration messages, with multiple readings from a node formed into a 32-byte payload with a preceding 4-byte header to identify the node concerned. Telemetry start and stop are directed by the user on the PDA application.

### 2.3. Mobile Device Application

The application on the user's PDA is written in C# for the Windows Mobile.net platform to allow the user to acquire, store, upload, download, and display medical readings. The data acquisition can come from the master node via Bluetooth and also by spreadsheet user entry for standard instruments. The data is stored on a small database on the mobile device to give secure and searchable access for the different data files. An Internet connection to the server allows a file to be searched for and uploaded or downloaded. Incoming master node telemetry, together with stored or downloaded spreadsheet image or waveform files, can also be displayed.

For the PDA to acquire data from the master node over the Bluetooth link, pairing is necessary, which is explicitly effected with the hard-coded PIN code of the master node and with the PDA as master. The PDA initiates the Bluetooth connection, allowing the node network to be configured and data telemetry to be started and stopped. 

To allow users to enter readings from standard instruments (i.e., without communications facility) and also to record the patient's general health, spreadsheets are used relating to cardiac and diabetic monitoring. Specifically, these values are entered by the user, selecting from the discrete value options of multiple list boxes. Instead of allowing the user to enter arbitrary values, such as in [3], which allowed speech and web form data entry, responses are constrained to certain values or ranges. Discrete selections allow some commonality between patient responses and trends can be more easily identified over a series of sessions. List boxes also allow the user to point and select, rather than type explicitly on a small or virtual keypad, and were also found to be a convenient method for a mobile phone [4]. Our colour coding on general health responses also gives a visual indication of the general condition of the patient. Text areas are also available for users to type notes if required. One drawback of a small screen and the number of parameters being monitored is the multiple pages a user has to navigate, so further work is ongoing to distil the information into a more concise representation of the patient's condition. 

**Figure 2:**
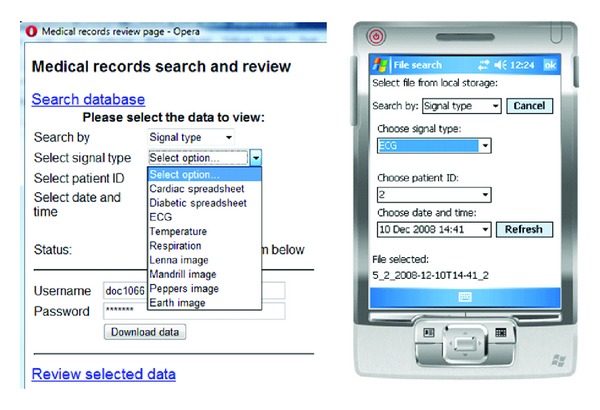
Example of database search on browser (left) and PDA application (right).

Multiple measurement files from different sessions and patients are stored on the PDA using Microsoft SQL Server Compact 3.5 [24], which is a secure and searchable database for desktop and mobile devices. Using search fields, the user can select a particular file to upload to the server or display on the PDA. Similarly, the user can search the MySQL database at the server to download a file over an Internet connection. The database search criteria used to distinguish the files are patient identification number, timestamp (date and time), and signal type (e.g., ECG, cardiac spreadsheet) which populate the search list boxes. These boxes are actively updated with database results as the search is narrowed and will show options such as all measurements for a particular patient and on a particular date and time. An example search is shown in Figure 2.

The incoming telemetry from the master node can be displayed on the PDA as well as spreadsheet, image, and waveform files read from local storage or from the server. The waveform and image displays can also be zoomed, scrolled, and manipulated by user controls to view specific regions as required.

### 2.4. Mobile Web Browser Controls

The available controls on the system's web pages are designed to provide a consistent interface to that of the PDA application, so the spreadsheet data entry, database search and display of downloaded spreadsheet, image and waveform files are very similar. Since the web pages can be viewed on PCs and the small screens of mobile devices, collapsible regions of the web page allow the user to show or hide relevant sections to reduce the amount of scrolling required.

The browser cannot acquire Bluetooth telemetry but, once logged in, the same spreadsheet interface as on the PDA application for standard instruments and general health can be used. This data cannot be stored locally so is uploaded to the server in the same logged-in session. Previous sessions are downloaded using the database search facility, which also uses list boxes for patient identification number, timestamp, and signal type as search criteria. These are actively populated from the server database over an Internet link as the search progresses. The selected spreadsheet, image, or waveform file is then downloaded and displayed similarly to the PDA application. These displays are generated by the browser to allow zoom, scroll, and manipulation, using controls on the web page in a similar way to the PDA application and independently of the server. 

### 2.5. Recorded Health Parameters

Besides being a means to store the measurements from standard instruments, spreadsheet dialogues were developed to find the most practical parameters to monitor for patients with cardiac and diabetic complaints. Practicing medical clinicians supported the project team at the All India Institute of Medical Sciences (AIIMS) and Aligarh Muslim University (AMU) and they were consulted to provide the most appropriate parameters and to give ongoing feedback during application development regarding the most appropriate symptoms for cardiac and diabetic monitoring. A further constraint was the limited screen size of the PDA for expressing questions and obtaining data input.

The data entry is a mixture of textual data, such as name and address and a list of measurements from a discrete list of possible responses, as shown in Table 2 for cardiac monitoring and Table 3 for diabetic monitoring. The diabetic data also includes a calculation of body mass index and waist-hip ratio. These parameters were not included in the cardiac monitoring as they were not identified for inclusion by the clinicians consulted. For diabetes the measurements selected are a subset of those taken during regular patient monitoring at the AMU Oncology Department.

Further research is required on trend analysis to obtain a profile of a patient's condition, as obtained with standardised health surveys such as SF-36 [25] and EQ-5D [26] for monitoring the effectiveness of treatment. The SF-36 and its variants, including SF-12 and SF-8, are a short-form survey of 36, 12, and 8 questions, respectively, designed to produce an overall set of scores for defined fields in a health profile. Similarly, the EQ-5D survey is an alternative measure and can calculate a single value for comparison. The trend analysis is important to monitor patient progress over time and may give early warnings of potential complications that may develop.

**Figure 3:**
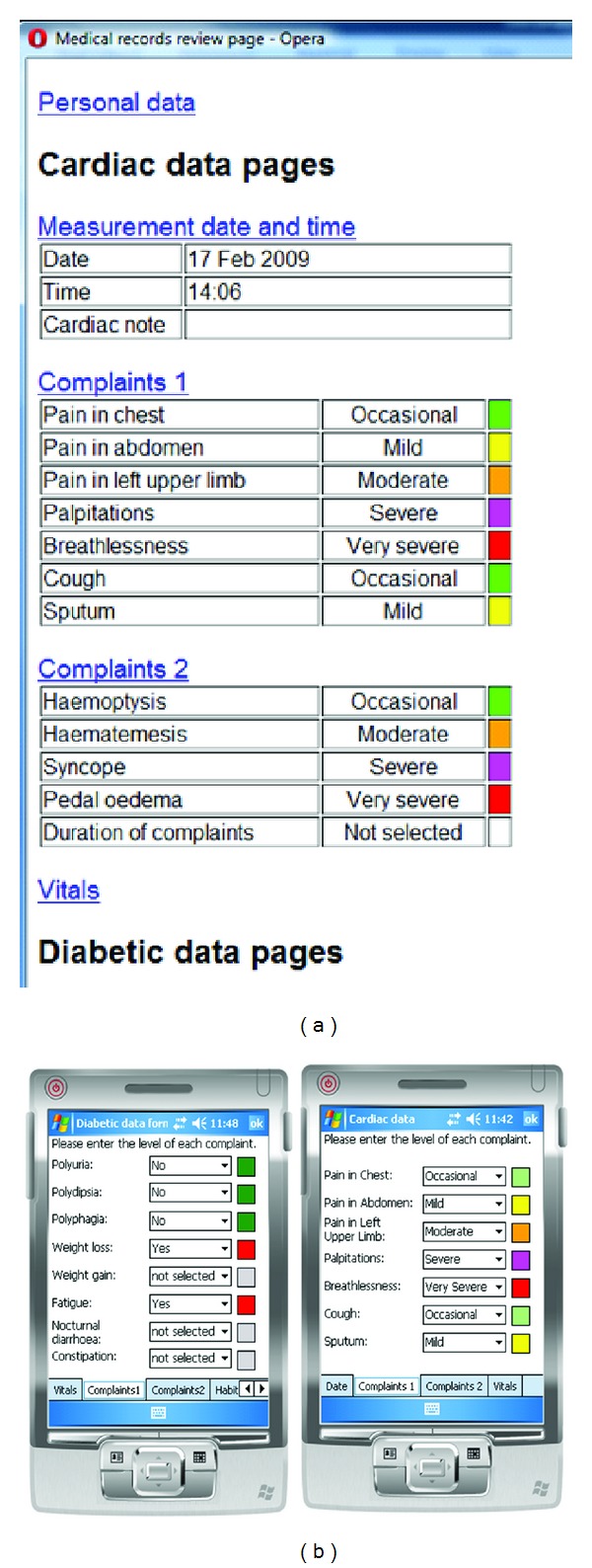
Example of spreadsheet data entry on browser (left) and PDA application (middle and right).

One issue with collecting a range of measurements is how to display this succinctly, rather than to read pages of responses. Our initial method is to code discrete values with colours, ranging from green for good to red for bad. A possibility to try to distil the whole response into a general overview of the patient's condition is currently in progress. An example spreadsheet entry is shown in Figure 3.

One common drawback in structured data gathering approaches is ensuring there is sufficient flexibility to allow the system to be tailored to the requirements of individual clinicians. The somewhat unsatisfactory solution taken (in this work as well as many others) is to allow free text input at some point of the consultation so that additional information or notes can be recorded. 

### 2.6. Medical Data Presentation on a Web Browser

Due to the requirement for an Internet connection to the server and the need for a cross-platform solution, a web browser is used for accessing patient data [1]. The main concern is the capability of the browser to generate the web page content, since there are two ways to generate web pages: “server-side” and “client-side.” Server-side scripting uses scripting languages (e.g., PHP, ASP, JSP) on the server to generate the HTML and any images or diagrams, and then download the content for the browser to display. Client-side scripting uses scripting languages (e.g., JavaScript, VBScript) on the user mobile or PC to generate some content instead.

Generating images and diagrams on the client-side has not been possible until recently without requiring the use of controls such as Microsoft's ActiveX, Java applets, or plug-ins such as Adobe Flash. The same constraint also applies to manipulating server-generated images, since the browser inherently reconstructs the image from the downloaded image file, so the underlying pixel data is not made available for client-side processing. To preserve the cross-platform approach and not require a specific operating system, Java environment or plug-in, the only alternative has been to generate the web page content on the server. For instance, to scroll or zoom into an image or waveform plot, the server would generate the required view or plot the required waveform part as an image and download it as a JPEG or PNG file for the browser to reconstruct and display. 

Previous studies into displaying medical data on mobile device browsers have found that the display of tables, images, and waveforms on small screens can be of high quality. The browser has been found to be a good platform for displaying tables and images, but for data manipulation a software application written for the device was used instead. An example of sending an image to a PDA browser is the display of a single-lead ECG contained in a PNG image [27]. In this case the web page was refreshed every 2s to display the next time window of the waveform. Another example is of server-generated web pages formatted by the server to suit the screen size of a test mobile device and the browser allowed a one-step zoom into images [28]. This allowed tabular data and images to be well presented on the device, but to display a multilead ECG on a mobile's small screen was found impractical [29], so the waveforms had to be separated. To enable this level of control required a C++ application on the PDA, rather than a browser.

Among our tests was a 12-lead ECG displayed as four groups of three leads, but it was found that scrolling a longitudinal ECG waveform display was not acceptable, so the waveform was also divided into 2.5 s windows shown at full screen width. To emulate this on a browser each “page” was downloaded separately. To reduce the number of pages downloaded, some systems had algorithms to optimise the amount of information per page. For large datasets, such as tabular test results, the pages were divided according to the device's screen size [30]. Adjacent pages were obtained by using hyperlinks to renew the display. Similarly, tabular data was split into “semantic blocks” and image data into subimages on the server, which then transmitted the first part [31]. For the tabular data, an optimising algorithm was used to reduce the amount of scrolling and searching needed to find results [32]. This was based on medical priority to decide which tabular results should be sent first. In this case also, to view the full image with scrollbars and zoom required an “Image Viewer” application to be written for the device.

Now, using some asynchronous JavaScript and XML (Ajax) [33] and HTML5 controls, this display manipulation can be made on the browser independently of the server and may remove the need for an application. Two newer client-side controls used in our system to give more dynamic control to the user's browser, especially a mobile browser, are the JavaScript XMLHttpRequest (XHR) object, as used in Ajax, and the HTML5 canvas element. The former is used in our system to search the server database and also to download image content, waveform samples and the cardiac and diabetic spreadsheet data for JavaScript to use. The latter provides the means to draw, display, and manipulate the spreadsheets, waveform diagrams and images dynamically with client-side scripting.

Canvas is new to HTML5 and is implemented in current releases of browsers such as Firefox, Safari, Opera, and Chrome, but not Internet Explorer 8 or below. XHR is supported on all these browsers. Currently Opera is the only browser supporting both the desktop and multiple mobile platforms with good support for Ajax and HTML5. It also includes the Dragonfly debugger to debug web pages in Opera running on a desktop or a mobile device [34]. Our system was tested with Opera Mobile 10 [35] on the PDA and the desktop browsers mentioned above.

#### 2.6.1. XMLHttpRequest

XMLHttpRequest [36] is used to access some specific data from a server, which could be a complete web page or just some information, without refreshing the whole page. For example, some search engines use this to suggest previous search phrases matching the query as the user is typing. In our database search web page, criteria for searching for a particular patient's file are sent and results are obtained dynamically using this method to narrow the search to a particular file. This file (spreadsheet, image, or signal) is then downloaded, also using this method. 

#### 2.6.2. HTML5 Canvas Element

The canvas element [37] allows images and pictures to be dynamically generated by JavaScript using a drawing “context” object. This context exposes methods for drawing lines and shapes onto a bitmap before displaying it on-screen. The bitmap pixel data can also be returned in an array of red, green, blue, and alpha values of each pixel, allowing some image processing to be performed before returning back to the canvas element for redisplay. Methods for image manipulation include a transformation matrix and selected area copy, allowing the image to be moved and scaled.

#### 2.6.3. Ajax File Download

The current XHR object specification [36] returns the server response as a Unicode string, so the file contents have to be transmitted in a suitable format and parsed in JavaScript (a future release [38] will also return the response as a byte array, simplifying the process). We tested three formats to download the files: a JavaScript object notation (JSON) comma-separated-value (CSV) format, a hexadecimal CSV format and as a binary string.

The JSON [39] format translates integer and floating point numbers into a decimal ASCII CSV string. For example, the values −32768, 0.2, and 32767 become “−32678, 0.2, 32767.” Hexadecimal representation is not allowed in a JSON string and is only appropriate for integer values, but the CSV string may be shorter. The values −32768, 0, and 32767 become “−8000, 0, 7FFF” in this format. In both cases JavaScript has native functions to parse the string into a numerical array.

To transmit the binary string requires more processing in JavaScript, but the downloaded file is no larger than the original and may be considerably smaller than the other formats. In this case the HTTP headers of the server response need to be set to prevent the bytes being interpreted as 7-bit text. These are set as “Content-Type: text/plain; charset=x-user-defined” and “Content-Type-Encoding: binary.” This allows each byte in the byte string to pass through as 8 bits and become a Unicode character in the XHR Unicode string. The bytes can then be extracted and recombined into C-style number types such as short or long as required. 

To compare the size of a downloaded file in each of the three formats, we transmitted short values from −32768 to 32767, a total of 131072 bytes. The resulting lengths are shown in Table 4 with the CSV formats at least twice as long as the byte string. Hence for integer values we use the byte string format and for floating point the JSON style.

#### 2.6.4. Canvas Plotting

**Figure 4:**
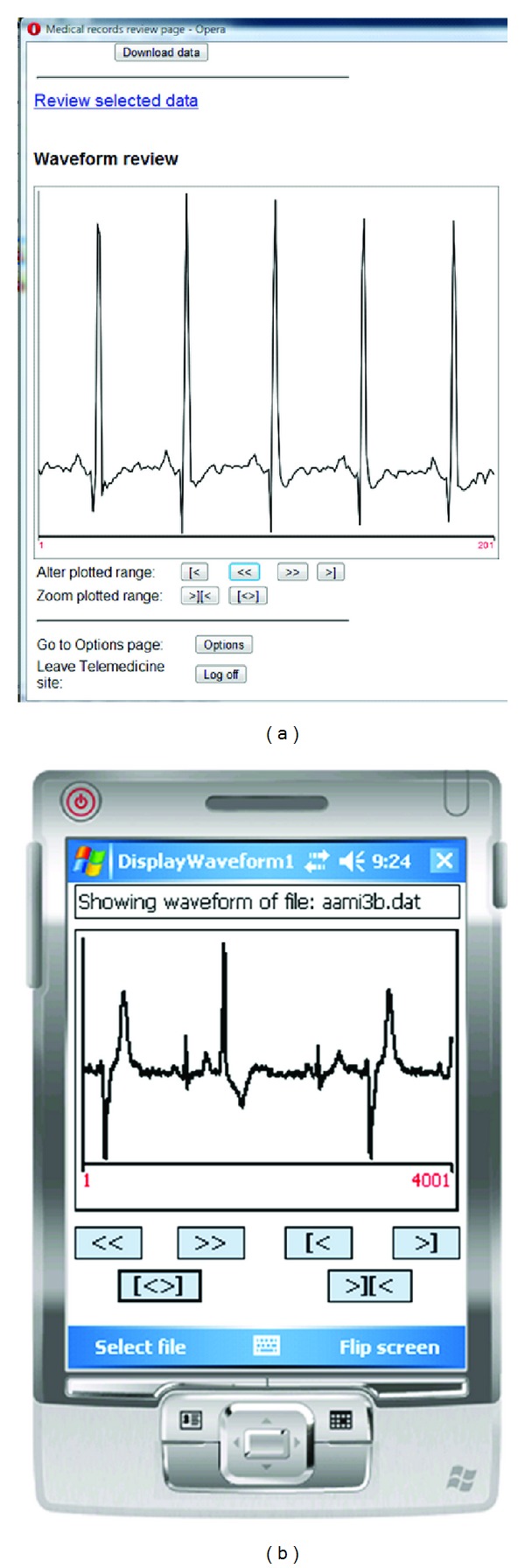
Example of waveform display on browser (left) and PDA application (right).

Once the data have been downloaded, dynamic plotting in the canvas element allows browser control of how information is displayed and can replace the applications required previously. It can generate charts, graphs and alter images based on controls operated on a mobile device without contacting the server. For instance, image pixels can be filtered to hide or highlight certain regions, with implications for medical image processing such as for X-ray image enhancement. An example ECG waveform display is shown in Figure 4.

### 2.7. Data Security and Watermarking

To ensure patient confidentiality, national regulations exist to address control of access and transmission of medical data with which telemedicine systems may have to comply. For instance, the U.S. Health Insurance Portability and Accountability Act (HIPAA) Part 164.312 includes “Security Rules” that specify both the appropriate verification of the identity of users and the prevention of access to transmitted data without authorisation [40]. Previous work has dealt with how these requirements can be delivered in telemedicine systems and over mobile telephone networks.

In terms of encryption of the transmitted data, the most usual solution is to use secure socket layer (SSL) or transport layer security (TLS) with a hypertext transfer protocol secure (HTTPS) web address [4, 5]. An alternative is to use a virtual private network (VPN) which effectively brings a remote user into the secure private clinic intranet over an insecure Internet link by a secure connection [32, 41, 42]. For mobile devices, an additional preliminary safeguard before making such a secure connection, or controling access to authorised users only beyond login details (e.g., username, password), is to verify the device used to access the data. The knowledge a mobile network operator has regarding its subscriber is used to verify the subscriber identity module (SIM) card to the clinic for data access [43, 44]. Once a connection is established and before data is transmitted, some additional mutual authentication between people at the remote ends is another safeguard proposed [45]. Here biometric data, such as from face recognition, was proposed to verify a clinician's identity to remote users. 

In our system we wanted to build on this previous work and help ensure the integrity, authentication, and identity of the data that had been received over these secure connections. In particular, we use digital watermarking techniques to embed pertinent information into the body of the data (signal, image, or document file) to aid its verification and also to aid monitoring [46]. Since the message is contained in the signal samples, image pixel values, and document character codes it is present within the file itself rather than in a header structure or a separate metadata file. Having such close coupling, watermarks are designed to be robust to unauthorised removal or tampering and such actions may result in the loss or damage of the original signal, image, or document. The premise is that if the watermark is applied at the point of acquisition, then it is present when the file is stored, transmitted, and displayed and the information contained can be used as another security safeguard. 

A large proportion of the published papers on digital watermarking apply it to digital rights management (DRM), which is intended to restrict the “acquisition, storage, processing, consumption, and export” of digitally delivered media [47]. As far as medical applications are concerned, some previous uses of watermarking have embedded records, including names and date of birth to identify the patient. In [48] textual data of a patient's name, date of birth and some medical history were embedded into an X-ray image and in [49] watermarks provided tamper detection, patient medical data, and physician information. Other examples include embedded medical Digital Imaging and Communications in Medicine (DICOM) header information into a radiograph image [50], patient reports or ECG segments into images [51] and digital signatures into images [52–54]. A digital signature is a summary or digest of the original file. If the original was an image for instance, the signature may be a known random sequence of values of the whole image. If the image is altered, then generating the same sequence and comparing it with the embedded sequence should indicate that the image has been altered. 

The embedded information in our system includes patient identification number, timestamp of the measurement, measurement type number, and hash values. The intention of the monitoring watermark is to use it as a general tool such as to embed annotation onto an ECG signal, possibly highlighting a problem area or as clinical notes. Subsequent access to the file finds and displays the annotation embedded.

In our implementation the verification watermarks are initially applied to the files at the patient device, since this is the point of acquisition. To store a file, the relevant patient identification number, timestamp, and signal type need to be entered before it can be allowed onto the database. Given this information, the watermark is created and embedded and the file stored in binary form. On accessing a file, the final search criteria (patient identification number, timestamp, and signal type) are cross-referenced against the embedded watermark. Similarly, before the server accepts a file upload, the intended patient identification number, timestamp, and signal type need to be supplied and can be checked at the server with the embedded watermark. Finally, for downloading from the server, the required search criteria are known by the receiver and are checked against the watermark. Assuming that the requested file is the one received and no tampering has been detected, the signal can be displayed with any annotation watermarks located.

## 3. Results

Since the partners in the UKIERI project comprise five institutions in England and India, each had work packages to develop and evaluate related to their respective interests: the sensor network and telemetry protocols at the Indian Institute of Technology Delhi (IITD), the cardiac spreadsheet format at AIIMS and the related diabetic spreadsheet at the Oncology Department of AMU, data security and trend analysis at AMU, the PDA application and database access at Loughborough University and diabetes monitoring at Kingston University, following on from their related work of diabetes monitoring using mobile devices [55, 56]. A series of group workshops has evaluated the complete system as the implementation has progressed. 

**Figure 5:**
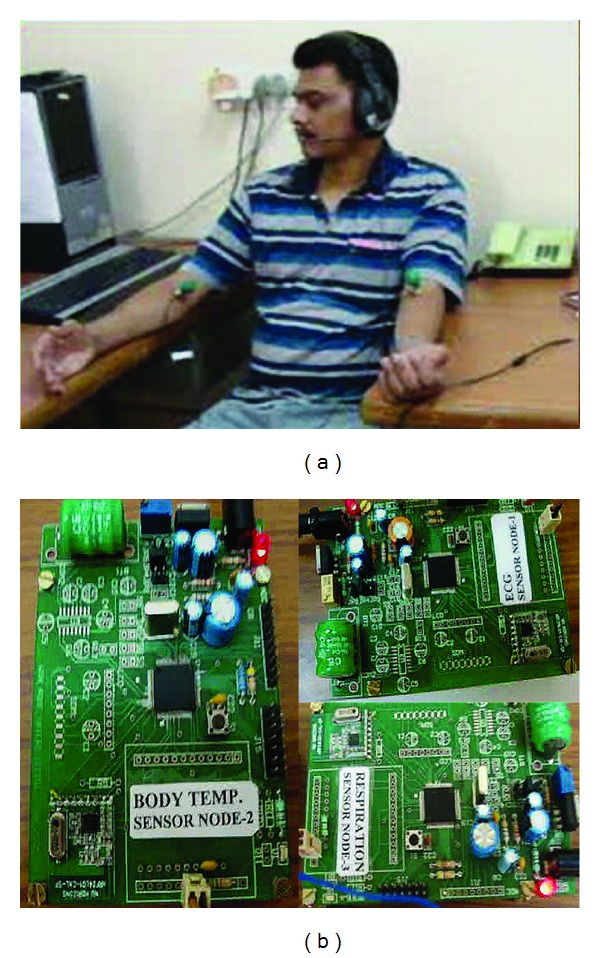
A patient's vital signs being recorded with three sensors measuring ECG, respiration and temperature.

A previous workshop sensor demonstration is shown in Figure 5, where a patient was monitored by three slave nodes for ECG, respiration, and temperature, respectively. The transducers consisted of Ag/AgCl suction cup sensors for the ECG, a thermocouple for the body temperature (oral or aural measurements were obtained), and a microphone to pick up the differential pressure to sense inward and outward breaths for respiration rate. The telemetry was acquired by the PDA and an Internet link was established between IITD and the server in England to successfully upload and download data over the mobile and Wi-Fi networks.

The forthcoming clinical evaluation is for field trials with patients and clinicians at AMU and AIIMS and their satellite rural clinics, which is one of the deployment scenarios specifically targeted.

## 4. Discussion

In this paper we have looked at our implementation of a system to monitor cardiac and diabetic conditions and highlighted the areas we found could be enhanced from previous systems by using alternative components or techniques. The inclusion of wireless and standard instruments gives a broadband approach to the diversity of parameters measured, and new fields, such as the wrist pulse analysis, may supply further insights into monitoring these conditions. 

In the evaluation and technical trials the system architecture was able to acquire, transmit, and display all the information we had decided to monitor in our clinical needs study. Now, with the system working, some further work is required on the data presentation, since this is the part clinicians will be interacting with most. We have seen how a clinician can download some information and dynamically change how this is displayed, but it remains to be seen which of the many parameters being monitored will be found most informative in practice, along with the preferred method of presentation. In the longer term, trend analysis using the measurements recorded would need to determine which are required and how they can be used in a health profile. 

## Figures and Tables

**Table 1:** Selected characteristics of some available 2.4 GHz transceivers.

Type	Bit rate (kbps)	MAC protocol	Node limit	Hops
ZigBee [12]	250	IEEE 802.15.4	2^64^	Multihop
ShockBurst [13]	1000 or 2000	Commercial	6	Single hop
ANT [14]	1000	Commercial	2^32^	Multihop

**Table 2:** Patient spreadsheet measurements for cardiac monitoring.

Parameter measured	Discrete values available
Temperature (°F)	96, 97, 98.6, 99, 100, 101, 102, 103, 104, 105
Pulse rate (bpm)	<60, 60–65, 65–70, 70–75, 75–80, 80–85, 85–90, >90
Systolic BP (mmHg)	80, 90, 100, 110, 120, 130, 140, 150, 160, 170, 180, 190, 200, 210, 220, 230
Diastolic BP (mmHg)	0, 10, 20, 30, 40, 50, 60, 70, 80, 90, 100, 110, 120, 130, 140
Respiration rate (brpm)	<16, 17, 18, 19, 20, 21, 22, 23, 24, 25, >25
Glasgow Coma Scale	
Eye response	0, 1, 2, 3, 4
Verbal response	0, 1, 2, 3, 4, 5
Motor response	0, 1, 2, 3, 4, 5, 6
Chest pain; abdominal pain; left upper limb pain; palpitations; breathlessness; cough; sputum; haemoptysis; haematemesis; syncope; pedal oedema	None, occasional, mild, moderate, severe, very severe
Duration	Not selected, up to 1 hour, 1–6 hours, 6–72 hours, >3 days, >2 weeks

**Table 3:** Patient spreadsheet measurements for diabetic monitoring.

Parameter measured	Discrete values available
Blood sugar (mg/dL)	80, 90, 100, 110, 120, 130, 140, 150, 160, 170, 180, 190, 200, 210
Pulse rate (bpm)	<60, 60–65, 65–70, 70–75, 75–80, 80–85, 85–90, >90
Systolic BP (mmHg)	80, 90, 100, 110, 120, 130, 140, 150, 160, 170, 180, 190, 200, 210, 220, 230
Diastolic BP (mmHg)	0, 10, 20, 30, 40, 50, 60, 70, 80, 90, 100, 110, 120, 130, 140
Respiration rate (brpm)	<16, 17, 18, 19, 20, 21, 22, 23, 24, 25, >25
Polyuria; polydipsia; polyphagia; weight loss; weight gain; fatigue; nocturnal diarrhoea; constipation; postprandial fullness; dyspnea; angina; loss of libido; cataract; diplopia; edema; sweating abnormalities; H/O viral episode	Not selected, no, yes
Alcohol; smoking; meals; sweets; exercise	Numbers are used to represent, respectively, the number of alcohol units, cigarettes, meals taken, sweets eaten, and hours of exercise (including walking, gardening, and climbing stairs)
Osmotic symptoms; incidental glycosuria; recurrent infections; nonhealing wounds; intense pruritus	Not selected, up to 1 hour, 1 to 6 hours, 6 to 72 hours, over 3 days, over 2 weeks
Blood sugar fasting/PP; urine albumin/microalbumin; serum creatinine; HbA1c; fasting lipid; hemogram; fundus	Discrete ranges for each parameter
Body mass index and waist-hip ratio	Discrete ranges for each parameter

**Table 4:** Comparison of downloaded content length for three data formats.

XHR data format type	Data content-length (bytes)
Byte string	131072
JSON CSV	403767
Hex CSV	351714
